# Heat loss evaluation for heating building envelope based on relevance vector machine

**DOI:** 10.1371/journal.pone.0314822

**Published:** 2025-06-18

**Authors:** Jianwei Yue, Jing Lu, Yang Yue, Yuqin Pan

**Affiliations:** 1 School of Civil Engineering and Architecture, Henan University, Kaifeng, China; 2 School of Civil Engineering, Xinyang Vocational and Technical College, Xinyang, China; 3 University College London, Gower Street, London, WC1E 6BT, United Kingdom; 4 Henan Academy of Building Research Co., Ltd, Zhengzhou, China; Agricultural Sciences and Natural Resources University of Khuzestan, IRAN, ISLAMIC REPUBLIC OF

## Abstract

Due to the influence of many factors, there is no reasonable evaluation method for the heat loss evaluation of the envelope, which leads to the deviation of the evaluation results of building energy consumption. By comparing different regression analysis models and considering the heat transfer characteristics of the envelope and heat loss evaluation needs, the dynamic reference value of heat loss evaluation “temperature baseline” which meets different energy saving standards is established. The evaluation method of instantaneous heat loss of the envelope is proposed and the heat loss is studied. The results showed that the performance indexes of relevance vector machine (RVM) regression model was superior to the other two regression models(response surface methodology (RSM), support vector machine (SVM). Based on the RVM regression model, the dynamic prediction model of “temperature baseline” was established by combining numerical simulation with regression analysis. Taking a residential building as an example, the envelope temperature baselines meeting the energy saving standard of 75% and 50% were obtained by using the prediction model, the temperature baselines of walls and windows that save 75% energy were −0.6°C and 1.9 °C respectively, and those that save 50% energy were 1.2°C and 4.5°C. The infrared image and heat loss evaluation method found that 87% of the points exceeded the temperature baseline by more than 2°C, and the total heat loss of the wall with an area of 13m^2^ was 471W. The heat loss evaluation method based on RVM proposed in this paper can objectively evaluate the heat loss of building.

## 1. Introduction

Heating and cooling account for the largest proportion of a building’s total energy consumption. Global statistics show that cooling energy accounts for about 6% of total building energy consumption, while heating energy accounts for more than 30% of total building energy consumption [1,2], especially in cold areas. With the increasing emphasis on building energy consumption, active energy conservation policies have been adopted at the national and regional levels. Among them, the energy-saving renovation of the envelope has been an important focus, as it affects the energy consumption and comfort of the building as well as the response to external conditions [3]. Obviously, as more heat is transferred through the building envelope, more energy needs to be consumed to maintain a similar indoor air temperature [4]. Therefore, the envelope structure is the main cause of heat loss. The transformation of higher thermal performance standards for buildings has important practical significance for energy conservation. The most effective measures and technologies will depend on the results of the energy performance assessment of each building. However, due to the general lack of heat metering equipment in existing buildings, it is difficult to obtain data on a building’s heating energy consumption and to assess building heat loss. This paper centers on the heat loss evaluation method of the envelope structure, aiming to establish a heat loss evaluation benchmark and provide a basis for heat loss evaluation.

Currently, most of the studies are conducted using laboratory testing methods and numerical simulation software for modeling and analysis to study and evaluate the thermal performance and heat loss issues of different buildings or structures in terms of energy efficiency. For example, Zhao et al [5] estimated the heat loss through the thermal bridge by linear thermal transmittance based on the heat transfer characteristics of the thermal bridge and utilized COMSOL Multiphysics software to establish a two-dimensional heat transfer mode of the thermal bridge and supplemented it with python for automated simulation and data processing. Samini et al [6] quantitatively evaluated and compared a variety of passive heating strategies used in Turkish baths to mitigate envelope heat loss through thermal simulations of two typical cases, Yazd Khan Baths and Golshan Baths, by performing computational modeling and quantitative analyses using DesignBuilder V7.0.1.004 software. In addition to this, laboratory testing methods for the thermal performance of the envelope are relatively mature, with the more commonly used methods being the heat flow meter method [7] and the hot box method [8]. However, these two methods are based on point and localized measurement and evaluation methods, and cannot accomplish comprehensive testing and evaluation of the target to be tested. Relying on the use of indoor and outdoor heat flow densitometers and temperature sensors as calculation inputs to determine thermal properties, obtaining these measurements can be restrictive, expensive and time consuming. In contrast, obtaining temperature measurements using an infrared camera is readily available and fast.

Regression analysis is widely used for prediction in various construction projects, including thermal performance of envelopes [9], material flexural properties [10], seismic fragility analysis of bridges [11], and values of thermal resistance in the Enclosed Airspaces of building envelopes [12]. Srinivasan et al [13] developed a prediction model for bend force and final bend angle (after springback) of air bending forming of electro galvanized steel sheet using RSM with strain hardening exponent, coating thickness, die opening, die radius, punch radius, punch travel, punch velocity as input parameters, and bend force and final bend angle as responses. The results obtained from the model fit well with the experimental results. In 2005, Dong et al. [14] first used SVM to predict the monthly energy consumption of four buildings in the tropics. The feasibility and applicability of SVM in the field of building load forecasting were tested, and the monthly average outdoor dry-bulb temperature, relative humidity and global solar radiation were used as three input features to predict building energy consumption in the tropics. Similarly, Zhong [15], Ding [16] et al. considered the high degree of nonlinearity between inputs and outputs and used support vector regression to predict building energy consumption and building cooling load. Jayaprakash et al [17] consolidated the compressive strength data of various self compacting concrete (SCC) mixes taking into account the effects of water cement ratio, water binder ratio and steel fibers. The applicability of regression based on RVM for predicting the compressive strength of various SCC mixes was explored. The predicted compressive strengths of SCC mixes were in good agreement with the corresponding experimental observations in the literature. Khedher [18] and Moayedi et al. [19] used artificial neural network to predict the heat loss of buildings based on the heat transfer coefficient of building walls and coating materials, as well as indoor, outdoor and external surface temperatures as input parameters.

Much of the current research is using infrared equipment to detect moisture [20–22], air leakage [23,24], and other defects [25–27]. Initially, thermal infrared methods were recognized as “qualitative methods”. The International Organization for Standardization (ISO) standard formulated in 1983 clearly states that infrared thermography can only qualitatively detect abnormalities in the thermal performance of building envelopes [28], and its quantitative use has not yet been investigated, and many problems remain unresolved. In other words, qualitative infrared thermography identifies anomalies, but it does not necessarily inform about the severity of the defects [29]. With the development of drones as well as infrared thermography equipment and technology, it has become possible to evaluate the heat loss of the building envelope. Therefore, more and more scholars at home and abroad have begun to study the application of thermal infrared methods for quantitative research.

Many scholars have utilized infrared imaging to evaluate heat loss in building thermal bridges. For example, Ascione et al [30,31] used infrared thermography as a supporting technique to visualize the location of the thermal bridge, using heat flow density measurements as a reference, and verified the reliability of the proposed simplified numerical procedure to efficiently and rapidly assess the heat loss of the thermal bridge. Benko [32] was one of the first researchers to assess heat loss through thermal bridging based on information collected from infrared images of building facades. Using an outdoor thermal camera, the surface uniform temperature of the undisturbed portion of the building envelope by thermal bridging, T_si_, and the surface temperature of the thermal bridge were recorded; using these two temperatures, Benk introduced an energy savings factor, ES, as the ratio of the heat loss of the building elements with and without thermal bridging. Similarly, Asdrubali et al [33] expressed the heat loss associated with thermal bridges in terms of a ratio, which reflects the increase in heat loss in the presence of thermal bridges through infrared thermography, by evaluating this ratio, i.e., the incidence coefficient I_tb_. As mentioned above, heat loss assessment of thermal bridges was implemented using a combination of infrared thermography and heat flow meter method measurements or calculations. Based on these studies, O’Grady et al [34] proposed a thermal loss assessment method for thermal bridges that is not supported by other measurements and evaluates the thermal bridge q_TB_ and Ψ-value, quantifies the thermal bridge heat flux rate and linear thermal transmittance using only infrared thermography.

**Fig 1:**
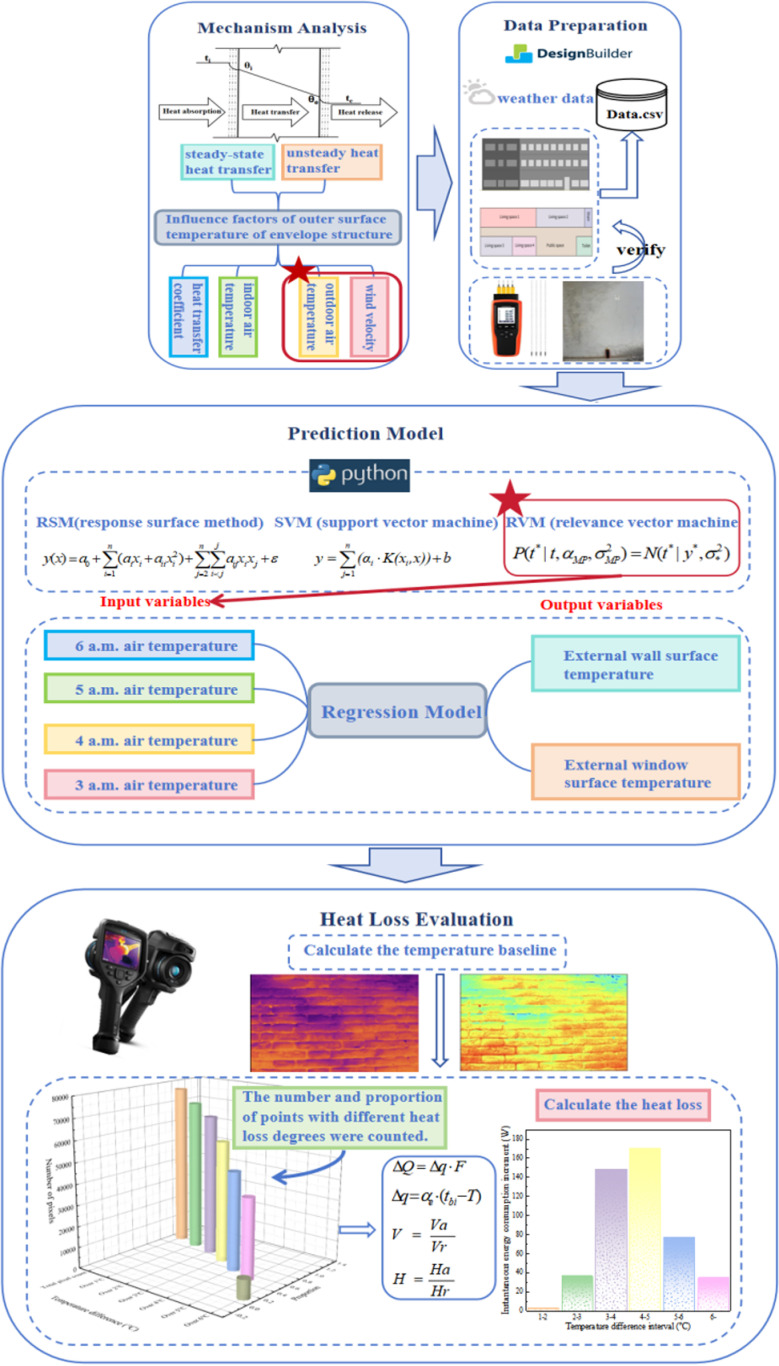
Flow chart of the proposed method. (Note: Red stars represent the key or final selection method.).

In summary, the heat loss evaluation methods of the envelope are gradually evolving from traditional laboratory testing and simulation to rapid testing means such as infrared thermography, which effectively solves the problem of difficult and slow statistics of energy consumption data. At present, most studies combine infrared imaging technology to study the heat loss of thermal bridges more, but it is difficult to study only thermal bridges to achieve a comprehensive analysis of building heat loss, mainly because of the lack of reference basis for heat loss evaluation. This paper proposes a transient heat loss evaluation method for envelope structures, which solves the problem of the lack of uniform standards for heat loss evaluation, establishes a heat loss evaluation benchmark “temperature baseline” using regression analysis, and quantitatively evaluates the heat loss of the envelope structure by combining infrared technology. The flow chart of the proposed method is shown in Fig 1.

## 2. Analysis of heat transfer characteristics of envelope

The heat transfer characteristics of the envelope directly affect the response and perception of its outer surface temperature, which in turn is related to the identification and solution of energy consumption and heat loss problems. The building envelope is constantly subjected to indoor and outdoor thermal actions [35–37], and heat is constantly transferred in or out through the envelope. Taking the wall as an example, in winter, the indoor temperature is higher than the outdoor temperature, and the main process of heat transfer of the envelope structure is: the inner surface absorbs heat from the indoor (mainly by thermal convection and thermal radiation); the heat transfers from the inner surface to the outer surface (mainly by heat conduction), heat transfer from the inner surface to the outer surface; the outer surface is oriented to outdoor heat release (mainly by thermal convection and thermal radiation) [38,39].

### 2.1 Steady heat transfer and unsteady heat transfer

**Fig 2:**
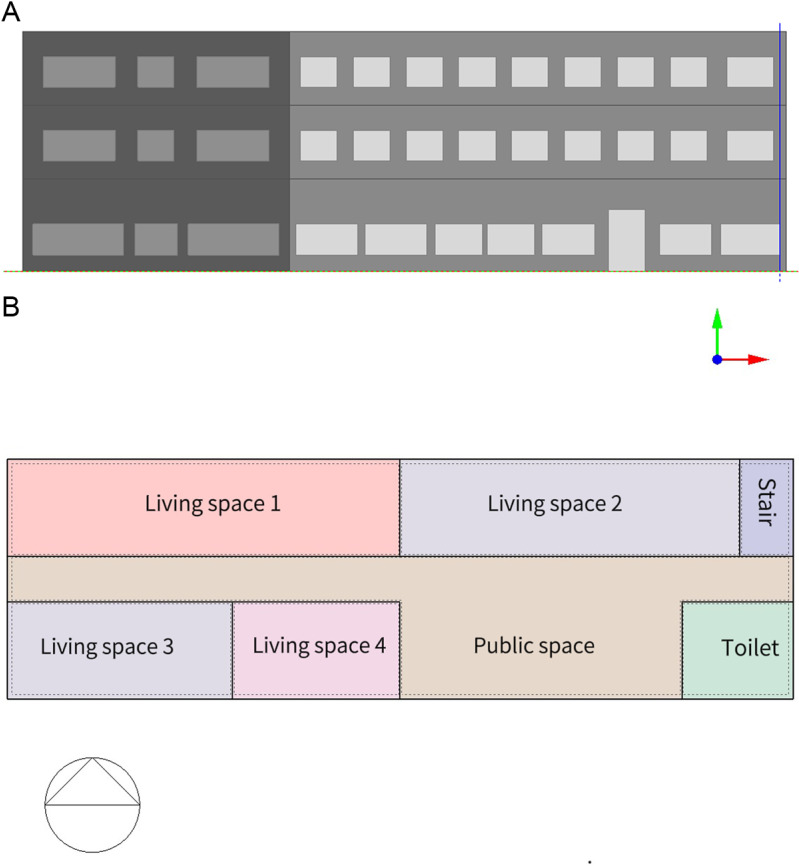
Unsteady heat transfer model of wall. (a) 3D model (b) Configuration.

The envelope structure with large heat transfer coefficient and small thermal inertia is most affected by unsteady heat transfer. In order to compare the outer surface temperature difference of the envelope structure corresponding to the steady heat transfer and the unsteady heat transfer, taking the typical external wall with poor thermal performance as an example, the unsteady heat transfer model is established by using Designbuilder software, as shown in Fig 2. Due to the different amount of solar radiation received by the envelope structure in different directions, the unsteady heat transfer process of the wall in different directions is not exactly the same. This paper takes the south facade and the north facade as an example to simulate the change of the outer surface temperature of the unsteady envelope structure with the outdoor air temperature.

**Fig 3:**
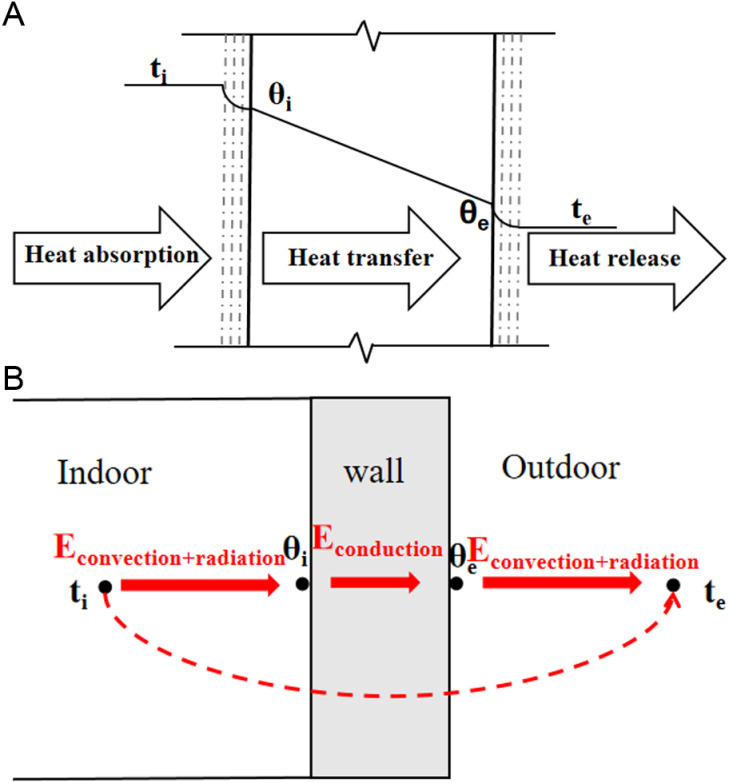
Steady heat transfer process of wall. (a)Heat transfer process of envelope structure (b) The basic way of wall heat transfer process.


For steady heat transfer, the heat transfer process can be decomposed into Fig 3. In the one-dimensional steady heat transfer process, Eq. (1) can be derived based on the energy balance equation:

$$K\cdot (t_{i}-t_{e})=E_{rad}+E_{conv}=\alpha_{r}(\theta_{e}-t_{e})+\alpha_{c}(\theta_{e}-t_{e})=h_{out}\cdot (\theta_{e}-t_{e})$$ (1)

where K represents the heat transfer coefficient of the envelope structure in $W/(m^{2}\cdot K)$, ti represents the indoor air temperature in °C, te represents the outdoor air temperature in °C, $\alpha_{r}$ is the outer surface radiation heat transfer coefficient in $W/(m^{2}\cdot K)$, $\alpha_{c}$ represents the outer surface convective heat transfer coefficient in $W/(m^{2}\cdot K)$, $\theta_{e}$ represents the external surface temperature of the envelope structure in °C, $\theta_{i}$ is the internal surface temperature of the envelope structure, $h_{out}$represents the external surface heat transfer coefficient, which is the sum of $\alpha_{r}$ and $\alpha_{c}$, and the main influencing factors are the external surface material, outdoor wind speed, etc. and the value is 23 $W/(m^{2}\cdot K)$.

**Fig 4:**
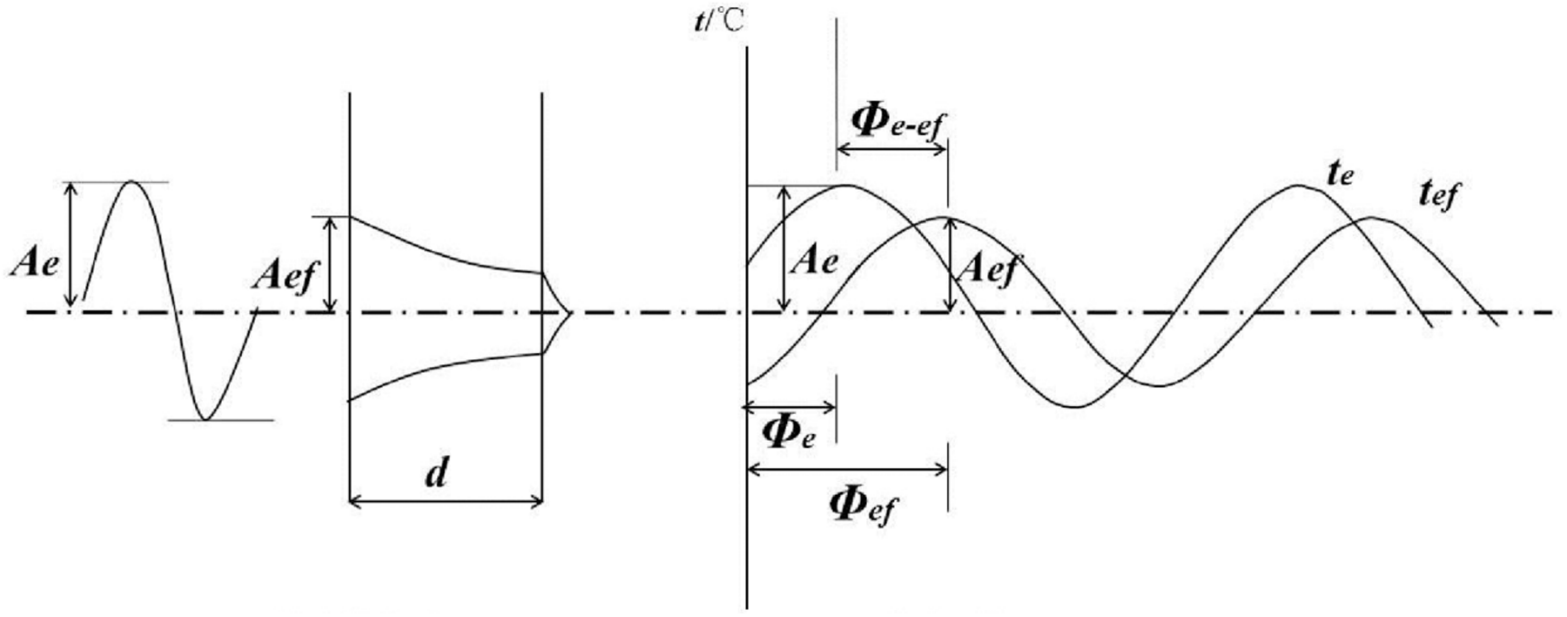
The temperature wave attenuation and phase delay when the harmonic action passes through the flat wall [40]. (a)Temperature wave attenuation (b) Phase delay.

Envelope structure by the thermal action of the indoor and outdoor environments, is changing with time, the heat transfer process is unstable heat transfer that is unsteady heat transfer, unsteady heat transfer can be simplified as periodic unsteady heat transfer, for the heating building, the indoor temperature is more stable in the winter, the outdoor air temperature shows a cyclical change, so the envelope structure is subject to pure harmonic displacement effect, as shown in Fig 4.

From Fig 4, it can be seen that both the air temperature and the outer surface temperature fluctuate with the same period, so it can be expressed as:


$$\begin{array}{c} \text{Outdoor temperature}\;\;t_{e}=A_{e}\cos(\omega \tau -\varphi_{e}) \end{array}$$ (2)

$$\begin{array}{c} \text{Outer surface temperature}\;\;t_{ef}=A_{ef}\cos(\omega \tau -\varphi_{ef}) \end{array}$$ (3)

where, $t_{e}$——Air temperature value at moment τ, °C;

$t_{ef}$——Outer surface temperature value at moment τ, °C;

$A_{e}$——Air temperature amplitude, °C;

$A_{ef}$——Outer surface temperature amplitude, °C;

ω——Angular velocity, $\omega =\frac{360}{Z}deg/h$, Z is the period of temperature wave, take 24h, ω=15deg/h;

τ——The time of temperature wave experienced, s;

$\varphi_{e}$——The initial phase angle of air temperature, °;

$\varphi_{ef}$——The initial phase angle of outer surface temperature, °.

The above equation shows that both the air temperature and the outer surface temperature fluctuate with the same period, except that the phase of the outer surface temperature is delayed for a period of time. In the steady heat transfer, the amount of heat transfer and the thermal conductivity of the material and the structure of the thermal resistance is closely related; for unsteady heat transfer, and the heat storage coefficient of the material and heat indolence. For example, a wall insulated in the cold outside environment. Assume that the thermal conductivity and thickness of the wall material are known and that the outside temperature remains constant. At this point, the internal and external surface temperatures of the wall remain stable for a certain period of time, and heat is transferred through the wall at a constant rate, forming a steady heat flow. In this case, the relationship between the heat flow and the temperature difference can be described by Fourier’s law, i.e., steady heat transfer. A wall illuminated by sunlight during the day. As the solar radiation changes, the temperature of the wall changes and the distribution of heat inside the wall changes over time. In this case, the temperature and heat flow between the inner and outer surfaces of the wall no longer remain constant, and heat will continue to accumulate and be released inside the wall. At this point, a dynamic analysis can be performed using the heat conduction equation to take into account the effect of time variation on heat flow, i.e., unsteady heat transfer. For whether the establishment of the temperature baseline proposed in this paper can simplify the unsteady heat transfer to steady heat transfer considerations, a study is needed to consider whether there is a large difference between the two modes of heat transfer on the outer surface temperature of the envelope.

**Fig 5:**
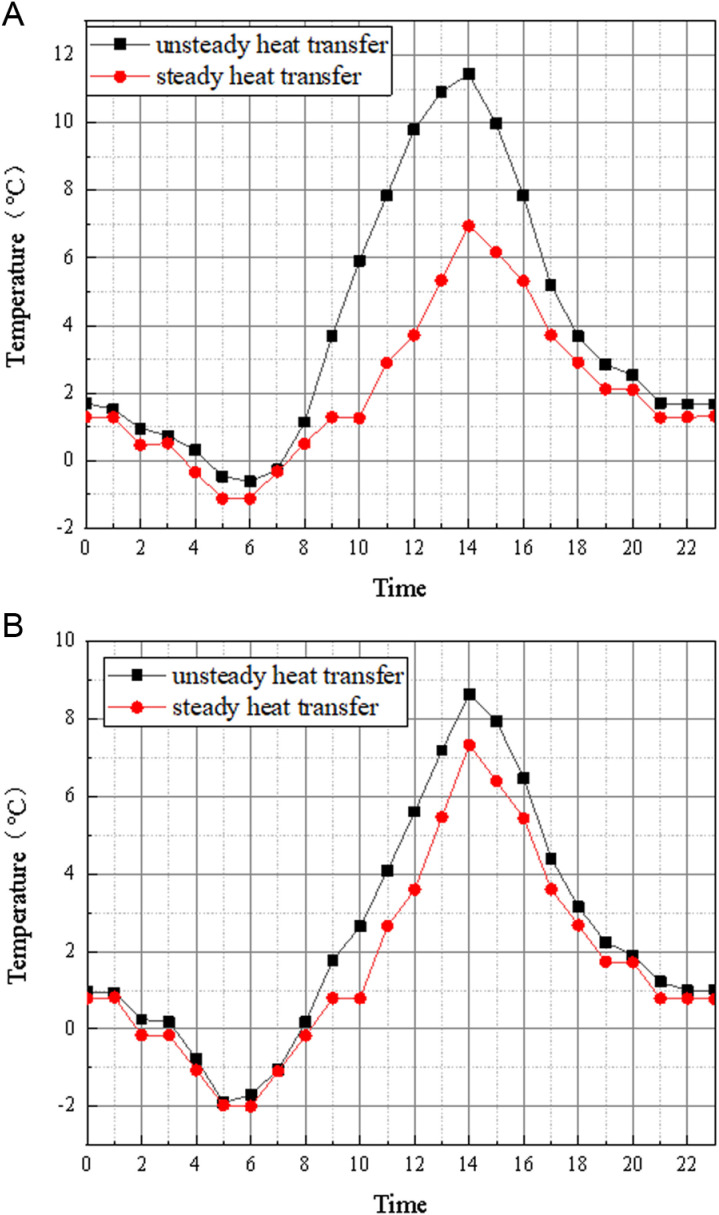
The change of outer surface temperature corresponding to unsteady heat transfer and steady heat transfer. (a) South facade (b) North facade.

For the unsteady heat transfer, keep the indoor temperature unchanged, take the typical outdoor temperature of Zhengzhou city as the meteorological parameter, simulate and calculate the unsteady heat transfer process and the change of the outer surface temperature; For steady heat transfer, the relevant parameters of the unsteady heat transfer model as well as the boundary conditions are substituted into Eq. (1) to calculate the 24-hour change of the outer surface temperature of the wall under steady heat transfer and without considering the influence of solar radiation, and the results are shown in Fig 5.

As can be seen from the above figure, for heating buildings, considering the characteristics of the envelope under steady state and unsteady heat transfer conditions, at night, especially during the time period 20:00–06:00 the next day, the difference in the temperature of the external surface of the envelope at the same moment is small, with a change of no more than 1 °Cat each moment. However, during the daytime, considering the steady and unsteady heat transfer of the envelope structure of solar radiation, the maximum surface temperature difference of the south wall can reach about 5 ~ 6 °C at the same time, and the temperature difference of the north wall also has a temperature difference of 2 ~ 3 °C. The results show that the unsteady heat transfer can be simplified to the steady heat transfer to study the heat loss of the envelope at night without considering the solar radiation. The results also give the best time period for infrared image acquisition as 20:00–06:00 the next day.

### 2.2 Factors affecting the temperature of the external surface of the envelope structure

For steady heat transfer, at night without considering the effect of solar radiation, the main factors affecting the temperature of the external surface of the envelope are heat transfer coefficient, indoor and outdoor air temperature and wind speed, and this section will focus on the study of the influence law of different factors on the outer surface temperature.

#### 2.1.1 Heat transfer coefficient.

In winter, the heat transfer coefficient of the outer surface is 23 $W/(m^{2}\cdot K)$, and the Eq. (1) is arranged into a functional relationship about the heat transfer coefficient, and Eq. (4) can be obtained:


$$\theta_{e}=\frac{\left(t_{i}-t_{e}\right)}{23}K+t_{e}$$ (4)

From the above formula, it can be seen that in the case of the same indoor and outdoor environments and wind speeds, the larger the heat transfer coefficient of the envelope, the higher the temperature of the external surface of the existing building. Further quantification of the effect of the heat transfer coefficient on the outer surface temperature reveals that the greater the temperature difference between indoor and outdoor, the more pronounced the test effect. For example, when the indoor-outdoor temperature difference more than 23°C and the heat transfer coefficient difference is 1 $W/(m^{2}\cdot K)$, the temperature change of the external surface of the envelope structure is more than 1°C; while when the indoor-outdoor temperature difference is less than 23°C and the heat transfer coefficient difference is 1 $W/(m^{2}\cdot K)$, the temperature change of the external surface of the envelope structure is less than 1°C. Therefore, when using infrared thermography to test the thermal performance of the envelope structure, the larger the indoor-outdoor temperature difference is, the more obvious the test effect is, and in general, it is necessary to ensure at least 5 ~ 15°C of indoor/outdoor temperature difference [41–46].

#### 2.1.2 Indoor air temperature.

Eq. (1) is organized into a functional expression of indoor air temperature, and Eq. (5) can be obtained:


$$\theta_{e}=\frac{K}{23}t_{i}+\frac{23-K}{23}t_{e}$$ (5)

From the above formula, it can be seen that for existing buildings with the same thermal performance of the envelope, the higher the indoor air temperature, the higher the outer surface temperature, given the same outdoor ambient temperature and wind speed. Further quantitative studies show that the effect of indoor air temperature on the outer surface temperature is very limited. When the difference in indoor air temperature is 1°C, the maximum difference in outer surface temperature of the external wall with poor thermal performance is about 0.13°C, and the difference of most external walls is below 0.1°C. When the difference in indoor air temperature exceeds 8 ~ 10°C, the difference in outer surface temperature is about 1°C. Therefore, for buildings with good thermal performance of the envelope, small changes in indoor air temperature can be considered as not affecting the outer surface temperature of the envelope.

#### 2.1.3 Outdoor air temperature.

From the above Eq. (5), it can be found that for buildings with the same thermal performance of the envelope, under the condition of the same indoor ambient temperature and wind speed, the higher the outdoor air temperature, the higher the outer surface temperature. For buildings with good envelope structure and thermal performance, $\frac{23-K}{23}$ is basically close to 1, and the change law of outer surface temperature is basically equivalent to the change of outdoor environment temperature. For buildings with poor thermal performance of envelope structure, such as single glass outer window, the outdoor air temperature changes by 1 °C, and the outer surface changes by about 0.7 °C, which is basically close to the change of outdoor temperature.

The outer surface temperature is strongly influenced by the outdoor air temperature. Therefore, when utilizing the thermal infrared method to study building heat loss, it is necessary to simultaneously record the outdoor ambient temperature corresponding to the time of infrared image acquisition, which is essential for quantitatively studying building heat loss.

#### 2.1.4 Wind speed.

The heat exchange between the outer surface temperature of the envelope and the external environment is an important heat loss pathway when the indoor and outdoor ambient temperatures are the same. Changes in wind speed have a significant impact on this process. Specifically, when the wind speed is high, the air flow will rapidly take away the heat emitted from the surface of the envelope structure, resulting in a decrease in the outer surface temperature. According to the basic principles of heat conduction and convective heat transfer, the rate at which heat is transferred from a heat source (building envelope) to a cold source (outside air) is directly proportional to the rate of air flow. Therefore, the greater the wind speed, the faster the rate of heat loss. As the wind speed increases, the temperature of the exterior surface gradually decreases, which is due to the fact that the larger wind speed enhances the convective heat transfer effect of the air, making the heat transfer between the surface of the envelope and the surrounding air more rapid. Wind speed affects the temperature of the outer surface of the envelope mainly by influencing the convective heat transfer coefficient [47], and the expression between the convective heat transfer coefficient and wind speed is as follows Eq. (6):


$$h_{out}=D+EV+FV^{2}$$ (6)

where D, E, F represent the roughness coefficient of the material surface, V is the wind speed in m/s.

In combination with Eq. (6), the above Eq. (1) is arranged into a functional expression of wind speed, and Eq. (7) can be obtained:


$$\theta_{e}=\frac{\left(t_{i}-t_{e}\right)}{D+EV+FV^{2}}K+t_{e}$$ (7)

For buildings with the same roughness and heat transfer coefficient of the outer surface of the envelope structure, under the same indoor and outdoor ambient temperature conditions, the greater the wind speed, the lower the corresponding outer surface temperature, that is, the heat lost through the envelope structure is quickly removed by convection. Therefore, the wind speed is an unfavorable condition, infrared image acquisition must strictly control the wind speed, should choose no wind or wind speed smaller environment for the actual measurement.

In summary, through the analysis of the heat transfer characteristics of the envelope structure, it can be seen that among the factors affecting the temperature of the external surface of the envelope structure, the heat transfer coefficient has a greater influence on the outer surface temperature, but the research object of this paper is for a single building, so that influence is no longer discussed; the influence of indoor air temperature is relatively weak and has its limitations; the outdoor air temperature and the wind speed, as the most important external factors affecting the external surface of the envelope structure, must be strictly controlled and the corresponding outdoor temperature at the time of image acquisition must be recorded when utilizing infrared equipment for the quantitative study of heat loss of existing buildings.

## 3. Heat loss evaluation benchmark

It is necessary to establish a detailed heat loss evaluation method for quantitative analysis of selected high heat loss buildings or a specific test building. In this paper, the concept of ‘ temperature baseline ‘ is proposed and defined as ‘ temperature baseline ‘: at a certain ambient temperature, the outer surface temperature corresponding to the envelope structure that meets different energy-saving standards is called the temperature baseline. The temperature baseline is the outer surface temperature of the envelope structure considering the influence of ambient temperature. This paper mainly focuses on walls and windows, and the difference between the temperature value obtained by infrared equipment and the temperature baseline can be used to quantitatively evaluate the heat loss of the existing building envelope and calculate the energy-saving retrofit potential of different buildings.

### 3.1 Necessity of establishing temperature baseline

According to the infrared picture, it can only quickly locate the location of temperature abnormality according to the temperature, and initially quantitatively determine the temperature difference between the temperature abnormality of the envelope structure and the overall temperature, and cannot quantitatively calculate the heat loss of the envelope structure from it. The ultimate goal of diagnosing and evaluating the heat loss situation of existing buildings is to propose the direction of retrofitting, which is to try to meet the requirements of local energy conservation standards. Therefore, it is necessary to study the distribution of the outer surface temperature of the envelope structure to meet different energy-saving requirements, so as to establish a reference temperature value for the comparison of the measured temperature, so as to judge the gap between the test building and the requirements of different energy-saving standards.

Currently, residential buildings in Henan have experienced four energy-saving standards of 30%, 50%, 65%, and 75% [48–51], and based on the definition of the temperature baseline, it is possible to establish the temperature baseline of the external surface of the envelope structure that is applicable to different energy-saving standards. For public buildings, according to the current national energy-saving standard level 72% [52], the public building temperature baseline (72% public building temperature baseline) can be established. Table 1 is the thermal performance index of the envelope structure corresponding to the temperature baseline meeting different energy-saving standards.

**Table 1:** Building energy efficiency standards implemented in Henan Province.

Standard naming	Heat transfer coefficient of the outer wall $W/(m^{2}\cdot K)$	Heat transfer coefficient of roof $W/(m^{2}\cdot K)$	Heat transfer coefficient of window $W/(m^{2}\cdot K)$	Energy saving rate
**(JGJ/26–1986) Civil architecture saving energy design standard (heating residential building part)**	1.5	1.0	5.5	30%
**(JGJ/26–1995) Civil architecture saving energy design standard (heating residential building part)**	1.0	0.8	4.0	50%
**(JGJ/26–2010) Design standard for energy efficiency of residential buildings in severe cold and cold regions**	0.7	0.45	3.1	65%
**(JGJ/26–2018) Design standard for energy efficiency of residential buildings in severe cold and cold regions**	0.45	0.3	2.2	75%
**(GB 55015−2021) General code for energy efficiency and renewable energy application in buildings**	0.45	0.3	2.2	72%

### 3.2 Method for establishing temperature baselines

Through the above study of the heat transfer characteristics of the envelope structure, it can be seen that, for the envelope structure with better thermal performance, the outer surface temperature is mainly influenced by the outdoor air temperature under the environment of a certain wind speed. Therefore, the establishment of the temperature baseline mainly studies the relationship between the outdoor ambient temperature and the outer surface temperature of the envelope structure.

On-site measurement of the outer surface temperature of the envelope is time-sensitive and environment-specific, with limited applicability, especially when establishing a “temperature baseline” that meets the energy-saving requirements, it is necessary to select a suitable existing building envelope to be measured, which increases the difficulty of implementation. The numerical simulation method calculates the outer surface temperature of the envelope by building a dynamic heat transfer model, which is advantageous in terms of high efficiency and controllability, but is limited by the complexity and uncertainty of the outdoor environment, which can only predict the temperature response under specific conditions and requires a long simulation time. Regression analysis models, on the other hand, are capable of exploring the mapping relationships of complex systems, providing mathematical expressions or computational models as transfer functions of system inputs and outputs [53]. The regression model not only predicts the temperature baseline under current conditions, but also generalizes to possible future scenarios, which effectively compensates for the limitations of numerical simulation alone to cope with complex environments and diverse input conditions. Therefore, combining numerical simulation and regression analysis methods, firstly, numerical simulation is used to obtain the sample data of the outer surface temperature of the envelope under different outdoor environments and energy-saving standards, and then regression analysis is utilized to establish the prediction model of the temperature baseline in order to efficiently and accurately obtain the target data.

### 3.3 Establish a database

#### 3.3.1 Simulation software.

DesignBuilder supports the simulation of multiple heat transfer modes, including conduction, convection and radiation, and is able to comprehensively evaluate the heat transfer characteristics of the envelope. The software can build a dynamic heat transfer model, which takes into account the heat transfer process of the envelope in different time periods, making the simulation results more accurate and realistic. In addition, DesignBuilder can use real meteorological data for simulation, and users can choose the appropriate meteorological data according to the actual situation, so as to simulate the heat transfer characteristics of the envelope more accurately. In summary, DesignBuilder has a variety of heat transfer modes and meteorological data support in heat transfer. Therefore, in this paper, Designbuilder software is used to simulate the heat transfer of envelope structures, so as to establish the database used for regression analysis.

#### 3.3.2 Validation of the suitability of the simulation software.

**Fig 6:**
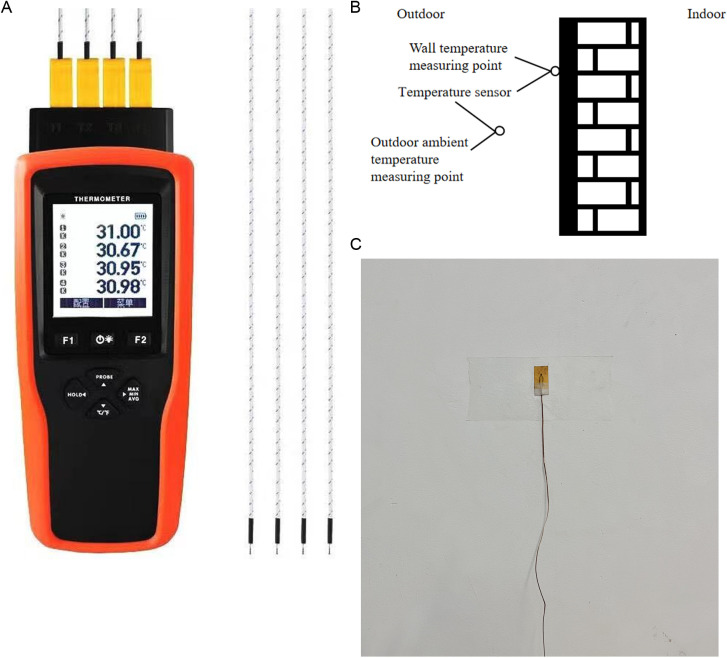
Sensor field installation. (a)Temperature recorder (b)Temperature measuring point position diagram (c)Thermocouple paste schematic diagram.

In order to verify the accuracy of the Designbuilder software in simulating heat transfer, the numerical model is now validated. Parameters such as personnel occupancy, equipment usage, and material properties associated with the envelope are entered into the Designbuilder software to match the actual situation as closely as possible. Since there is no meteorological data of Kaifeng in the software, the meteorological data of Zhengzhou city, which is the nearest to Kaifeng, is used for dynamic simulation, and the real meteorological data is used in validation. The four-channel temperature sensor (Fig 6(a)) was used to collect data on the outdoor air temperature and the outer surface temperature of the envelope structure (Fig 6(b)), the air temperature was measured by the temperature susceptibility wire, and the wall surface temperature was measured by the K-type surface paste thermocouple probe (Fig 6(c)), and the data were recorded once every half an hour, two of which recorded the air temperature, and the other two channels recorded the surface temperature, and finally the average value of each moment was taken. The simulation results were compared and analyzed with real data to assess the accuracy and reliability of the simulation software to determine the applicability of the simulation software, and the results are shown in Fig 6.

**Fig 7:**
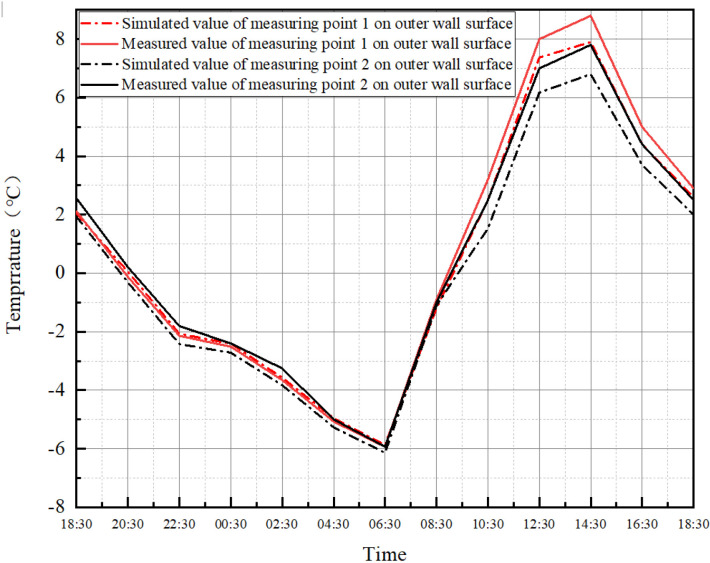
The temperature value of the outer wall measuring point.

As shown in Fig 7, the comparison results show that the simulated data are closer to the measured data, and the maximum error is about 1°C. For the simulation of the wall surface temperature, the error during the day is larger than that at night, and the maximum error occurs at the moment of the highest temperature. This is due to the fact that the outer surface temperature of the wall is affected by solar radiation during simulation, and this paper uses the default solar radiation intensity value of the software, which is not taken into account in the actual measurement. Therefore, there will be some errors between the measured and simulated values. However, in this study, the test time is at night and the wall is not subjected to solar radiation, so it is considered that the software can more accurately simulate the outer surface temperature of the envelope structure in the actual environment.

#### 3.3.3 Simulation to obtain sample data.

For the energy-saving envelope structure, the outer surface temperature is not greatly affected by the indoor temperature, but mainly affected by the outdoor temperature. The establishment of temperature baseline can not consider the influence of indoor temperature, and the indoor temperature can be set as the minimum value of indoor heating design temperature of 18 °C.

Due to the thermal stability of walls, the wall surface temperature at a given moment is influenced not only by the current outdoor environment, but also by the outdoor environment of the previous hours. Therefore, calculating the outer surface temperature of the envelope at a given moment according to the instantaneous temperature has limitations. The dynamic algorithm proposed in this paper for predicting the outer surface temperature at a certain moment in time takes into account not only the effect of the current temperature on the outer surface temperature of the envelope, but also the effect of the outdoor ambient temperature of the previous hours on the outer surface temperature of the envelope.

Before determining the simulation sample data, it should be determined that the outdoor ambient temperature of the previous few hours will have an impact on the outer surface temperature of the wall at a certain time. In this paper, the Designbuilder software is used to simulate the change law of the outer surface temperature of the energy-saving wall.

**Fig 8:**
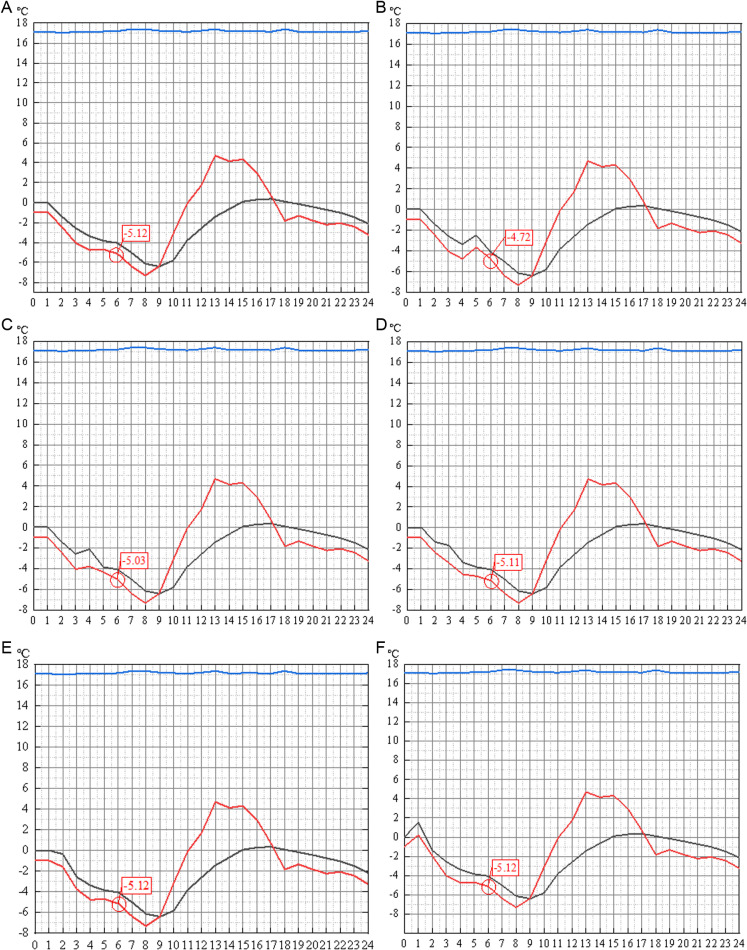
Changes of wall surface temperature under different outdoor temperatures.

At night, when there is no solar radiation, and in an environment with no wind or low wind speeds, the outdoor air temperature does not change abruptly at adjacent moments, and the rate of change of the outdoor air temperature is considered to be less than 2 °C during the time period in which the infrared thermography is in use. Take the outer surface temperature of the wall at 6:00 a.m. as the comparison temperature, denoted as T_0_, keep the outdoor air temperature t_0_ unchanged at the moment of 6:00 a.m., and change only the 05:00 outdoor air temperature t_-1_, 04:00 outdoor air temperature t_-2_, 03:00 outdoor air temperature t_-3_, 02:00 outdoor air temperature t_-4_, 01:00 outdoor air temperature t_-5_, respectively. The corresponding outer surface temperatures of the wall at 06:00 are simulated and calculated respectively, denoted as T_0_, T_-1_, T_-2_, T_-3_, T-_4_, and T_-5_, as shown in Fig 8.

Through Fig 8, it is found that the outdoor air temperature at the moment of 05:00 and the outdoor air temperature at the moment of 04:00 have some influence on the outer surface temperature of the wall at the moment of 06:00, with the influence values of 0.40°C and 0.09°C, respectively, and the air temperature at the moment of 03:00 has a small influence on the wall surface temperature at the moment of 06:00, with the influence value of only 0.01°C, which can be ignored. The outdoor environment at 04: 00 and the previous moments had no effect on the outer surface temperature of the wall. The results show that for the wall that meets the energy-saving requirements, the outer surface temperature of the wall at a certain time at night is only affected by the outdoor air temperature at the moment and the first three hours. Therefore, in the regression analysis performed below, the outdoor air temperature at the moment of 06:00 and for the first three hours is used as an input variable, and the resultant parameter of the study is the temperature of the outer surface of the envelope.

### 3.4 Comparative analysis of temperature baseline prediction models

Although there are many statistical methods and machine algorithms for prediction, this study selectes specific methods (RSM, SVM, RVM) to predict the outer surface temperature of the envelope, mainly for the following reasons: Regression analysis based on RSM can solve the problem that the relationship between the objective function and the input independent variable is unknown, and can be used to model and analyze the problem that the objective function is affected by multiple variables to optimize the objective function. SVM is a popular supervised learning algorithm widely used for classification and prediction tasks. SVMs can handle nonlinear patterns and produce accurate predictions for energy data. SVMs have proven to be one of the most powerful data mining techniques [54]. RVM [17,55] is a sparsification model obtained in data training based on Bayesian principles applied to regression problems. It is similar to the functional form of SVM and is an extension of SVM, both of which solve nonlinear problems by introducing kernel functions and increasing dimensions, among others. Compared with traditional SVM, RVM has more flexibility and generalization ability, and usually has better performance when dealing with high-dimensional data.

Since each algorithm has its advantages, the choice of a specific algorithm depends on the characteristics of the data and the goal of the prediction application. In this paper, taking the 50% and 75% temperature baseline of residential building energy efficiency standards as an example, more than 600 sets of envelope outer surface temperatures at different ambient temperatures obtained by simulation are polynomially fitted and trained, and the outdoor air temperatures of four consecutive hours are used as the input variables, and the envelope outer surface temperatures at the moment of 06:00 are used as the response variables. Three regression analysis methods, RSM, SVM and RVM, were used for predictive analysis, and the prediction models corresponding to the different algorithms were implemented using computer programs, which were written in the python language and used the corresponding libraries and frameworks in the program environment. The dataset obtained from the simulation was divided into training and test sets, 90% of the sample data was taken for fitting and training for all three methods, and 10% of the sample data was used to test the performance of the model and evaluate the model prediction effect. In order to accurately predict the outer surface temperature of the envelope, it is important to consider the feasibility of the regression model. The temperature baseline prediction model is determined based on the characteristics and distribution of the data, the number and type of features, and other factors.

#### 3.4.1 RSM regression analysis.

*3.4.1.1 RSM mathematical model:* The purpose of this study is to predict the temperature of the outer surface of the envelope by means of the regression prediction model. Therefore, the temperature of the outer surface of the wall and the outer window is selected as the response quantity in the RSM. The mathematical model established by the RSM is essentially a mathematical polynomial model with a specific order. The expression is as follows Eq. (8):


$$y(x)=a_{0}+\sum_{i=1}^{n}\left(aix_{i}+a_{ii}x_{i}^{2}\right)+\sum_{j=2}^{n}\sum_{i≺j}^{j}a_{ij}x_{i}x_{j}+\varepsilon$$ (8)

where $a_{0},a_{i},a_{ii},a_{ij}$ is the fitting coefficient of the regression model, n represents the number of variables.

**Fig 9:**
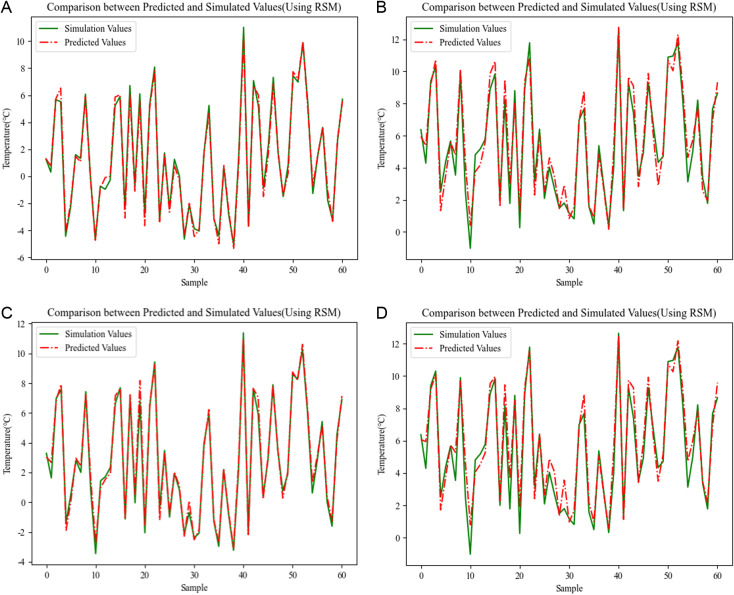
Comparison between predicted and simulated values(Using RSM). (a) Energy saving 75% wall prediction results (b) Energy saving 75% window prediction results (c) Energy saving 50% wall prediction results (d) Energy saving 50% window prediction results.

*3.4.1.2 Results of RSM regression analysis:* When using RSM regression analysis model for nonlinear prediction analysis of the outer surface temperature of the envelope structure is limited to the computational complexity of high-order polynomial fitting, this paper only gives the second-order nonlinear prediction model based on the RSM regression analysis, Fig 9 illustrates the prediction effect of the model in predicting the temperature of the outer surface of the envelope structure.

Observing the prediction results for the outer surface temperature of the wall in Fig 9, it can be seen that the polynomial fitting regression analysis method based on RSM achieves good fitting results when fitting the prediction analysis for the outer surface temperature of the wall. The trends of the predicted and simulated values are basically in agreement, and the error increases only in a few cases, with the maximum error being about 0.5°C. Observing the prediction results for the outer surface temperature of the window in the figure, it can be seen that the fitting effect of the polynomial fitting regression analysis method based on RSM for fitting the outer surface temperature of the window for prediction analysis is not as ideal as that of the external wall, and the prediction error is larger, up to a maximum of about 1.5°C. Therefore, it is considered that the RSM-based prediction method cannot realize the effective prediction of the outer surface temperature of the external window, and its prediction accuracy stability is poor in multiple sample scenarios.

The drawback of the RSM polynomial fitting method lies in the computational complexity of the higher-order function. The above analysis only considers the second-order coupling cross-effects between the influencing factors and ignores the complex higher-order coupling effects of the temperature response to the outer surface temperature for four consecutive hours. Although the computational complexity is avoided, it also has a certain impact on the prediction performance of the regression model. Therefore, in order to more fully learn the mapping relationship between each influential parameter and the response variable, SVM regression analysis and RVM regression analysis methods are introduced to predict the outer surface temperature of the envelope.

#### 3.4.2 SVM regression analysis.

*3.4.2.1 SVM mathematical model:* SVM is a supervised machine learning technique. It proves to be useful for solving multiple regression and classification problems. SVR is commonly used in prediction problems and the general model is expressed as Eq. (9):


$$y=\sum_{j=1}^{n}\left(\alpha_{i}\cdot K(x_{i},x)\right)+b$$ (9)

where y represents the predicted value; x represents the input vector; $x_{i}$ represents the training data vector; $\alpha_{i}$ represents the Lagrangian coefficient; $K(x_{i},x)$ represents the kernel function for calculating similarity between $x_{i}$ and x; b represents the offset value.

**Fig 10:**
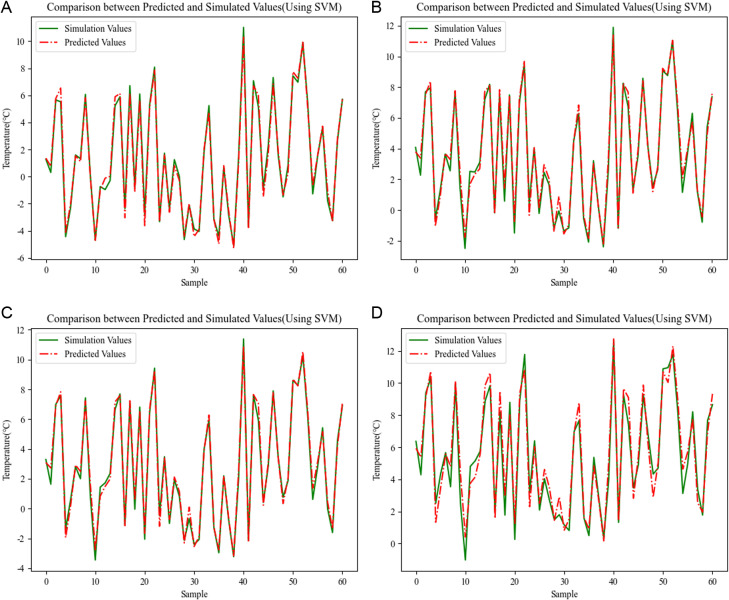
Comparison between Predicted and Simulated Values(Using SVM). (a) Energy saving 75% wall prediction results (b) Energy saving 75% window prediction results (c) Energy saving 50% wall prediction results (d) Energy saving 50% window prediction results.

*3.4.2.2 Results of SVM regression analysis:* Fig 10 illustrates the prediction effect of the model in predicting the outer surface temperature of the envelope, which are the prediction results of the outer surface temperature of the wall and the outer surface temperature of the window obtained based on the SVM regression model, respectively. With the SVM regression analysis method, the wall prediction results are well fitted, and the errors between the predicted and simulated values are basically in the range of [−0.2°C, + 0.2°C], and the prediction results in individual scenarios are only increased, with the maximum error of about 0.5°C or so. The prediction results of the outer window show that the accuracy of the fitting prediction analysis based on the SVM regression analysis method is significantly lower than that of the outer wall. The overall trend of the predicted value of the outer surface temperature of the outer window is basically consistent with the simulated value, but the error fluctuation of the two is obvious, and the maximum error of 1 °C, which reflects the lack of prediction stability of the SVM-based regression analysis prediction method. To compensate for this deficiency, a RVM-based regression analysis method is introduced to predict and analyze the temperature of the outer surface of the envelope.

#### 3.4.3 RVM regression analysis.

*3.4.3.1 RVM mathematical model:* The RVM is a model that utilizes a weighted combination of kernel functions applied to a regression problem. Let the learning sample data set be $\left\{X_{n},t_{n}|n=1,..\mathrm{.2},N\right\}$. Establish the relationship between $X_{n}$ and $t_{n}$*:*


$$t_{n}=y(x_{n};\omega )+\xi_{n}$$ (10)

$$y(x,\omega )=\sum_{n=1}^{N}\omega_{n}K(x,x_{n})+\omega_{0}$$ (11)

where $K(x,x_{n})$ is the kernel function, $\omega =\{\omega_{n}{\}}_{n=0}^{N}$ represents different weight value types, $\xi_{n}$ represents the additional Gaussian noise satisfying $\xi_{n}\backslash \;N(0,\sigma^{2})$. Because the Gaussian kernel function is stable and has strong linear interpolation ability, this paper introduces the Gaussian kernel function:


$$\begin{array}{c} K(|y-y_{c}|)=\exp\left\{-\frac{|y-y_{c}{|}^{2}}{2\delta^{2}}\right\} \end{array}$$ (12)

where $y_{c}$ is the kernel function center, and δ is the Gaussian kernel width.

Assuming that $t_{n}$ is independent distribution, the likelihood function of the learning sample is:


$$p(t|\omega ,\sigma^{2})=(2\pi \sigma^{2}{)}^{-N/2}\exp\{-\frac{1}{2\sigma^{2}}||t-\Phi \omega |{|}^{2}\}$$ (13)

where $t=(t_{1},...,t_{n}{)}^{T}$, $\omega =[\omega_{0},\omega_{1},...,\omega_{N}{]}^{T}$, Φ is a N×(N+1)-order matrix. The Bayesian perspective method is applied to the RVM training model, and the weight $\omega_{n}=0$ is set to form a simple function about ω.


$$P(\omega |\alpha )=\prod_{n=0}^{N}N(\omega |0,\alpha_{n}^{-1})$$ (14)

It is assumed that the Gamma distribution of hyperparameters α and noise parameters satisfies $\sigma^{2}$:


$$P(\alpha )=\prod_{n=0}^{N}Gamma(\alpha_{n}|a,b)$$ (15)

$$P(\sigma^{2})=Gamma(c,d)$$ (16)

where $Gamma(\alpha |a,b)=\Gamma (a{)}^{-1}b^{a}a^{a-1}e^{-ba}$, $\Gamma (a)=\int \infty_{0}t^{a-1}e^{-t}dt$, in order to obtain more uniform hyperparameters, a=b=c=d=0 is generally taken. The probability distribution of the weight vector ω is:


$$P(\omega |t,\alpha ,\sigma^{2})=\frac{P(t|\omega ,\sigma^{2})P(\omega |\alpha )}{P(t|\alpha ,\sigma^{2})}=(2\pi {)}^{-(N+1)/2}|\sum {|}^{-1/2}\exp\{-\frac{1}{2}(\omega -\mu {)}^{T}\sum -1(\omega -\mu \}$$ (17)

where $\sum =(\sigma^{-2}\Phi^{T}\Phi +A)$ is the variance, $\mu =\sigma^{-2}\Phi^{T}t$ is the mean value, $A=diag(\alpha_{0},\alpha_{1},\ldots ,\alpha_{N})$ is the diagonal matrix. In the process of hyper-parameter training update, most of the values of weight ω are close to zero, and only a small number of sample points corresponding to non-zero weights play a role. Using this method to train samples can significantly reduce the number of model basis functions, so that the model can achieve sparse effect.

Through the above training to complete the RVM learning, in the iterative process to find the hyperparameters α and $\sigma^{2}$. The optimal values $\alpha_{MP}$ and $\sigma_{MP}^{2}$ are optimized based on the posterior distribution. The predicted values $t^{*}$ are distributed as follows:


$$P(t^{*}|t,\alpha_{MP},\sigma_{MP}^{2})=\int P(t^{*}|\omega ,\sigma_{MP}^{2})P(\omega |t,\alpha_{MP},\sigma_{MP}^{2})d\omega$$ (18)

where, in the calculation of the predicted probability distribution, the function is obtained by multiplying two Gaussian normal distributions, so the predicted distribution of T also obeys the Gaussian normal distribution, that is:


$$P(t^{*}|t,\alpha_{MP},\sigma_{MP}^{2})=N(t^{*}|y^{*},\sigma_{*}^{2})$$ (19)

where, the expected value is $y^{*}=\mu^{T}\varphi (x^{*})$, variance is $\sigma_{*}^{2}=\sigma_{MP}^{2}+\varphi (x^{*}{)}^{T}\sum \varphi (x^{*})$, the average value of the predicted value $t^{*}$ of the sample $x^{*}$ to be tested is distributed as $y^{*}=(x^{*};\mu )$.

**Fig 11:**
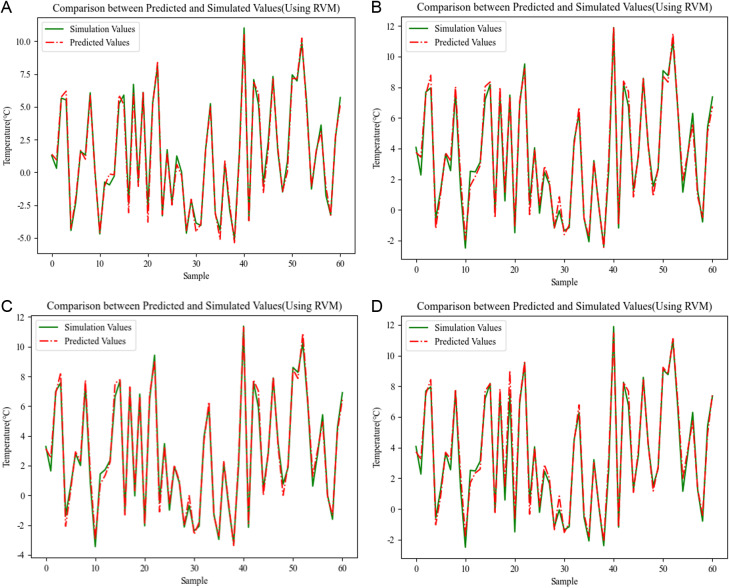
Comparison between predicted and simulated values(Using RVM). (a) Energy saving 75% wall prediction results (b) Energy saving 75% window prediction results (c) Energy saving 50% wall prediction results (d) Energy saving 50% window prediction results.

*3.4.3.2 Results of RVM regression analysis:* Fig 11 shows the prediction effect of the model on the outer surface temperature of the envelope. As can be seen from the above figure, good fitting results are achieved when responding to the outer surface temperature of the wall based on the RVM regression analysis model. The prediction calculation results for more than 60 test samples have a good match with the simulated values, the match is as high as 99%, and the error is basically in the [−0.2°C, + 0.2°C] interval, the maximum error is about 0.4°C or so, it can be considered that with the help of the RVM regression method can be based on the four consecutive moments of the point temperature response to achieve the goal of high-accuracy prediction calculations of the outer surface temperature of the wall. From the diagram, it can be seen that the predicted value of the outer surface temperature of the outer window is basically consistent with the simulated value. The error between the two was basically [−0.3°C, + 0.3°C], and the error fluctuation was not obvious, and the maximum error is 0.5°C. There is no significant large error calculation scenario, which reflects the stability advantage of RVM regression analysis method in long-term trend prediction.

#### 3.4.4 Optimal model for temperature baseline prediction.

Since each algorithm has its own advantages, the specific algorithm selection depends on the characteristics of the dataset and the goals of the prediction application. Two performance evaluation indexes, R^2^ and RMSE, are used to evaluate the quality of the regression model, and equations (20) and (21) explain the calculation of R^2^ and RMSE.


$$RMSE=\sqrt{\frac{1}{n}\sum_{i=1}^{n}{\left(yi-\hat{y}i\right)}^{2}}$$ (20)

$$R^{2}=1-\frac{\sum {\mathrm{\backslash nolimits}}_{i}{\left(yi-\hat{y}i\right)}^{2}}{\sum {\mathrm{\backslash nolimits}}_{i}{\left(yi-\bar{y}\right)}^{2}}$$ (21)

where n is the number of occurrences, $\bar{y},yi,\hat{y}i$ are the response variable’s average, estimated, and modelled quantities, respectively.

Table 2 gives the calculated results of the performance evaluation indexes for the three prediction models. As can be seen from the above table, the calculated values of R^2^ are all very close to 1, indicating that the models are able to explain the variability of the dependent variable (predicted values) very well, and that the fit between the predicted values and the simulated values is good, with no overfitting or underfitting. In addition, RMSE measures the square root of the average difference between the predicted and simulated values of the model, and the smaller the value, the higher the reliability of the prediction model. Comparing the values of RMSE of the three models, it can be seen that the value of RMSE of the prediction model based on RVM method is lower than the value of RMSE of the two prediction models based on RSM and SVM, i.e., RVM prediction model has the highest prediction accuracy. In general, the fitting effect of the three models for the wall is relatively good, but from the fitting effect of the window, it can be seen that the advantage of the RVM regression model is obviously higher than that of the other two models. Therefore, the RVM regression model is selected to predict the temperature baseline.

**Table 2:** Comparison of prediction performance indexes of different prediction models.

Forecasting model	R^2^	RMSE
**RSM**	0.973	0.0997
**SVM**	0.989	0.0623
**RVM**	0.994	0.0302

Measure the air temperature from 3:00–6:00 on a certain day with the Yuwen temperature sensor, as well as the outer surface temperature of the envelope at the moment of 6:00, input the air temperature into the model to predict the outer surface temperature of the envelope, and compare the predicted value with the measured value, and the results are shown in the Table 3.

**Table 3:** Comparison of predicted and measured results.

t_0_	t_-1_	t_-2_	t_-3_	Measured Value		Predicted Value								
						RSM			SVM			RVM		
				Twall	Twindow		Twall	Twindow		Twall	Twindow		Twall	Twindow
−3	−1.4	0.5	1.0	−1.1	3.3		-.1.0	2.5		−1.1	3.0		−1.1	3.2

From the above results, the predicted values of the three regression methods for the outer surface temperature of the wall are very close to the measured values, but the RSM and SVM prediction models are unstable for the windows, while the RVM prediction model is more accurate for the outer surface temperature of the windows, which further verifies the feasibility and accuracy of the RVM prediction model.

## 4. Heat loss evaluation method

For infrared images, the smallest element is a pixel point, and the difference between each pixel point in the image and the temperature baseline under the respective operating conditions can be quantitatively evaluated by comparing it with it on a pixel-by-pixel basis.

### 4.1 Prediction temperature baseline

According to the prediction method for the temperature baseline of the outer surface of the envelope to meet different energy-saving standards proposed above, the RVM-based prediction model is used to calculate the temperature baseline under different working conditions. The outdoor ambient temperature of each building at 6:00 a.m. and the first three hours was recorded by a Yuwen thermometer, which was used as the input data for the RVM prediction model to predict the temperature baseline corresponding to the conditions of infrared image acquisition for each test building, and the results are shown in Table 4.

**Table 4:** Test ambient temperature and temperature baseline of different energy-saving standards.

Building type	t_0_	t_-1_	t_-2_	t_-3_	Energy saving 75% temperature baseline		Energy saving 50% temperature baseline	
**Twall**	**Twindow**	**Twall**	**Twindow**
**Residential building**	0.6	1.4	1.7	2.6	−0.6	1.9	1.2	4.5

### 4.2 Quantifying heat loss

#### 4.2.1 The number and proportion of points with different heat loss degrees are compared and output pixel by pixel.

The shooting of infrared images is easily affected by climatic conditions: temperature, wind speed and solar radiation. In order to improve the shooting accuracy, the following measures are taken [56,57]: 1) place the infrared equipment outdoors half an hour in advance so that the infrared equipment is close to the outdoor temperature; 2) to avoid the influence of solar radiation, the time of shooting the infrared image is at night; and 3) in order to avoid the influence of the wind speed, the shooting environment should be in the absence of wind or the wind speed is small.

**Fig 12:**
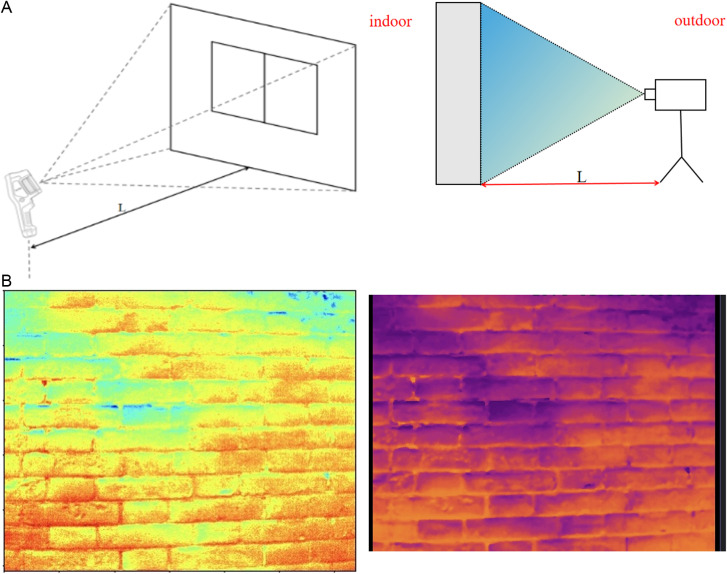
The infrared thermal imager captures the schematic diagram and infrared image of the envelope structure. (a) Shooting schematics (b) Original infrared images (left); Clear color image processed by python (right).

An infrared image of a wall or window is taken with the help of a FLIR E75 thermal imaging camera, schematically shown in Fig 12. An infrared image is usually stored in digital format, each pixel point corresponds to a temperature value, and the resolution of the captured image is 320 × 240, so an infrared image has 76,800 pixel points, corresponding to 76,800 temperature values.

**Fig 13:**
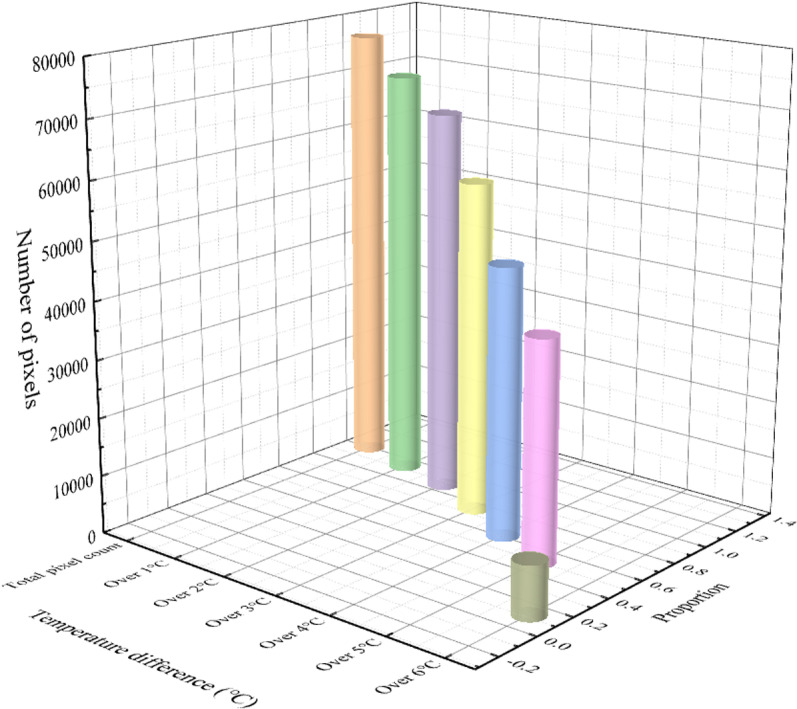
The number and proportion of pixels in each interval of the result of pixel-by-pixel comparison.

This section takes the external wall of a residential building as an example to elaborate the heat loss calculation method. The temperature of each pixel point is compared with the external wall temperature baseline of −0.64 °C, and the points with a temperature difference of more than 1 °C, more than 2 °C, more than 3 °C, more than 4 °C, more than 5 °C, and more than 6 °C are screened and counted, and the results are shown in Fig 13.

As can be seen from the above figure, the points where the temperature difference exceeds 6°C or more account for 12% of the total wall area compared to the 75% temperature baseline for energy efficiency in residential buildings; the points with a temperature difference of more than 4 °C account for 61% of the total wall; and more than 87% of the points have a temperature difference of more than 2°C from 75% of the temperature reference line (−0.6 °C).

#### 4.2.2 Calculate the energy saving potential of different heat loss degree.

For different degrees of heat loss, compared with the energy saving standard 75%, the heat loss amount, namely the energy consumption increment, is further quantified to determine the potential of its energy saving transformation. Denote the amount of heat loss as ΔQ, then there are:


ΔQ=Δq·F (22)

$$\Delta q=\alpha_{e}\cdot (t_{bi}-T)$$ (23)

where ΔQ represents the instantaneous heat consumption difference of the envelope structure in W, Δq represents the difference of heat flux intensity on the outer surface in W/m^2^, F represents the surface area in m^2^, $\alpha_{e}$ represents the heat transfer coefficient of the outer surface, here take 23 $W/(m^{2}\cdot K)$, $t_{bi}$ represents the infrared measured temperature of the outer surface in °C, Trepresents the temperature baseline in °C.

Therefore, to calculate the instantaneous heat loss ΔQ of the envelope structure, the local area should be calculated or measured first. According to the test distance and the parameters of the infrared thermal imager, the side length of the pixel can be calculated. Using the formulas (24) and (25), the length and width of a pixel can be calculated, and the area corresponding to different heat loss degrees can be calculated.


$$V=\frac{Va}{Vr}$$ (24)

$$H=\frac{Ha}{Hr}$$ (25)

where V represents the width of the pixel, $V_{a}$ represents the vertical field of view angle, and $V_{r}$ represents the vertical resolution, H represents the length of the pixel, $H_{a}$ represents the horizontal field of view angle, and $H_{r}$ represents the horizontal resolution.

The infrared resolution of the thermal imaging camera used was 320 × 240 and the field of view was 24° × 18°, so that the length and width of the image element was 1.3 mm, a square, and the area of each pixel was 0.000169 m².

**Fig 14:**
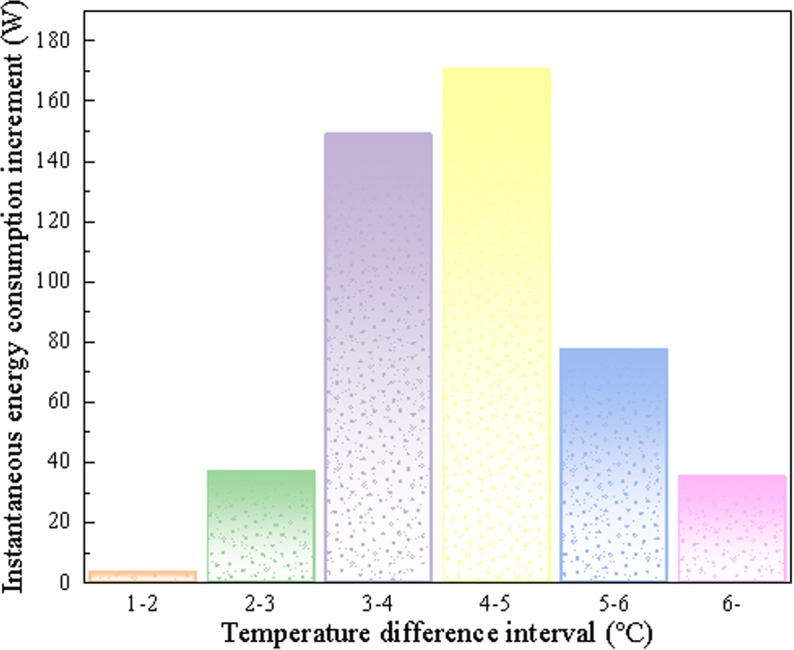
The amount of heat loss corresponding to different heat loss degrees.

Table 5 shows the area and heat loss of different temperature difference intervals. By quantifying the heat loss in different heat loss intervals, a more accurate evaluation of each part of the heat loss can be made. The table is drawn into Fig 14, and it is found that the most serious heat loss is in the area where the temperature difference exceeds 6 °C, and the heat loss is not the most. On the contrary, the area where the temperature difference is between 4–5 °C has the most heat loss due to the large area ratio. The results show that the amount of heat loss is not only related to the degree of heat loss, but also closely related to the area of heat loss. If conditions permit, energy-saving renovation should first be carried out in areas with a temperature difference of more than 3 °C to achieve the purpose of reducing energy consumption.

**Table 5:** Heat loss corresponding to different degrees of heat loss.

Different temperature range	F(m^2^)	ΔQ(W)
**Temperature difference greater than 1 °C less than 2 °C**	0.095	3.264
**Temperature difference greater than 2 °C less than 3 °C**	1.065	36.743
**Temperature difference greater than 3 °C less than 4 °C**	4.307	148.592
**Temperature difference greater than 4 °C less than 5 °C**	4.262	170.341
**Temperature difference greater than 5 °C less than 6 °C**	2.243	77.384
**Temperature difference greater than 6 °C**	1.012	34.914

In summary, the pixel-by-pixel comparison of the infrared test temperature with the temperature baseline enables a detailed heat loss evaluation of the test building façade, including locating the location of the severe heat loss; quantifying the severity of the heat loss; and calculating the amount of heat loss.

### 4.3 The key role and practical significance of heat loss evaluation method in the field of building energy conservation

Against the backdrop of the global climate change and energy crisis, energy saving and emission reduction have become a consensus among governments and societies. As one of the major areas of energy consumption, energy efficiency improvement in buildings is essential to reduce overall energy demand and minimize greenhouse gas emissions. Heat loss evaluation, as an effective tool, can help identify and quantify energy losses in buildings so that targeted measures can be taken. For example, in the energy efficiency retrofit of older homes, heat loss evaluation can identify areas of significant heat loss, such as windows and walls, so that targeted insulation and sealing measures can be taken; for large commercial buildings, heat loss evaluation can develop targeted improvement programs, such as optimizing air conditioning systems and adding high-performance glazing, to reduce overall energy consumption; and in the design phase of new buildings, heat loss evaluation provides architects with the data to help select appropriate materials and design layouts to reduce heat loss at the source. Specifically, through the heat loss evaluation, it is found that windows and external walls are the main heat loss points, and winter heating costs are higher than the average of similar buildings. Therefore, in the energy-saving renovation, high-performance double-glazed windows were selected and insulation was added to the external walls. After the retrofit, winter heating energy consumption was reduced by more than 30%, while the indoor temperature was maintained at a comfortable level. Energy efficiency not only has short-term benefits, but also has a wider impact in the long term. The popularization of energy-efficient buildings can drive the growth of the green building market and promote the development and innovation of related technologies and materials, ultimately creating a virtuous cycle.

The practical significance of heat loss evaluation in energy conservation and emission reduction is not only reflected in the reduction of building energy consumption and greenhouse gas emissions, but also promotes the sustainable development of the construction industry. Through the implementation of heat loss evaluation and corresponding improvement measures, it can make a positive contribution to the fight against global climate change. Such an in-depth discussion provides a solid theoretical foundation and empirical support for the paper, further emphasizing the importance of heat loss evaluation.

## 5. Discussion

The building sector accounts for about 40% of global energy consumption, with the envelope (e.g., walls, windows, and roofs) accounting for a significant portion of the energy consumption. Effectively evaluating and improving the heat loss of the envelope can significantly reduce building energy consumption. This paper proposes a method for evaluating the instantaneous heat loss of envelope structures, which uses regression analysis to establish a baseline “temperature baseline” for heat loss evaluation and combines infrared technology to quantitatively evaluate the heat loss of the envelope structures, which is more efficient, accurate and convenient than the traditional method, but the method also has certain limitations.Since the proposed method is based on infrared equipment and simulation, errors will inevitably exist despite the fact that methods such as environmental control and calibration equipment have been adopted to mitigate the uncertainties and major deviations in the results as much as possible. Since this paper mainly focuses on the local heat loss evaluation of heating buildings, in the future research for each facade of the independent part of the external walls and windows, pixel-by-pixel extraction and calculation of their measured temperature averages, the use of measured temperature averages and the “temperature baseline” of the difference between the value of Δt as a grading index for each facade of the external walls and windows, respectively, the overall heat loss grading. Finally, the instantaneous heat loss, ΔQ, is calculated for the walls and windows respectively, and the overall heat loss is further evaluated by ΔQ for each façade of the building. In addition, this paper mainly focuses on the climatic characteristics of cold regions, and the same method can be used to study buildings located in different climatic regions in future research. Heat loss evaluation can help us understand the energy efficiency of buildings and identify the main pathways of heat loss, so that we can optimize the design and improve the insulation materials to reduce energy consumption and improve occupant comfort, as well as help to reduce the environmental impact and operating costs.

Envelope heat loss evaluation plays a crucial role in building energy efficiency, especially in the context of addressing the global energy crisis and climate change. Through scientific evaluation methods, it can not only reduce building energy consumption, but also provide a solid foundation for the development of sustainable buildings. In the future, with the continuous advancement of technology, the envelope heat loss evaluation method will become more accurate, helping the building industry to develop in the direction of greener and more efficient.

## 6. Conclusions

In this paper, with the help of infrared imaging technology, a heat loss evaluation method applicable to heating buildings is proposed, which establishes a heat loss evaluation benchmark. On this basis, the heat loss of the envelope is quantitatively evaluated. The specific research work and conclusions are as follows:

(1) This study compares the difference between two heat transfer methods, steady state and unsteady heat transfer, on the outer surface temperature of the envelope. The research results show that during the day, considering the solar radiation, the outer surface of the south and north walls have a certain temperature difference, up to about 6°C, but at night, the temperature difference of the outer surface of the envelope at the same time is small, and the change of each moment does not exceed 1°C. Therefore, at night when solar radiation is not considered, the heat loss of the envelope can be studied by simplifying the unsteady heat transfer to steady heat transfer.
(2) In this study, heat loss evaluation benchmarks that satisfy different energy-saving standards are established. The three regression models of RSM, SVM and RVM are analyzed comparatively, and the evaluation indexes of RVM are better than the other two. Therefore, based on the RVM regression model, this paper establishes the dynamic prediction model of “temperature baseline” by the method of “numerical simulation + regression analysis”, and obtains the temperature baseline of wall and window that meet the energy saving standard of 75%, which are −0.6 °C and 1.9 °C respectively, and the temperature baseline of Twall and Twindow, which are 1.2 °C and 4.5 °C respectively to meet 50% of the energy-saving standard.
(3) With the temperature baseline as the reference temperature, the instantaneous heat loss of the envelope structure was calculated. 87% of the points exceeded the temperature baseline by more than 2 °C, and the total heat loss of the wall with an area of 13m^2^ was about 471W。

This study demonstrates that the transient heat loss of the envelope can be quickly quantified by establishing evaluation benchmarks that meet different energy-saving criteria and incorporating infrared technology. And in future research, further energy-saving retrofitting of existing building envelopes can be carried out based on the results of this paper.

## Supporting information

S1 File Database. (XLSX)
